# The effect of radiotherapy on survival of dental implants in head and neck cancer patients

**DOI:** 10.4317/jced.52346

**Published:** 2016-04-01

**Authors:** Bassam Shugaa-Addin, Hashem-Motahir Al-Shamiri, Sadeq Al-Maweri, Bassel Tarakji

**Affiliations:** 1 Department of Oral Maxillofacial Sciences, Al-Farabi Colleges, Riyadh

## Abstract

**Objectives:**

To explore the current literature of the survival of dental implants in irradiated head and neck cancer patients considering the role of implant location, bone augmentation, dose of radiation and timing of implant placement.

**Study Design:**

Pubmed search was conducted to identify articles published between January 2000 and December 2014 and presenting data of dental implant survival with radiotherapy in head and neck cancer patients. Studies on animal subjects and craniofacial implants were excluded.

**Results:**

18 articles out of 27 were eligible for inclusion in this systematic review. 12 out of 18 studies reported favorable outcome of dental implants and radiotherapy with survival rates between 74.4% and 97%. Seven out of ten studies comparing the survival rates according to site of implant placement reported that implants were found to osseointegrate with greater success in the irradiated mandible than irradiated maxilla. 5 studies which compared implant survival in irradiated native bone versus irradiated grafted bone reported that irradiated grafted bone showed a significantly reduced dental implant survival rate in comparison to irradiated native bone. 6 out of 18studies in which radiation doses exceeded 70 Gy reported lower survival rates of dental implants in comparison to the studies in which radiation doses were ≤70Gy. Higher survival rates were reported in 2 studies in which implants placement was before radiotherapy in comparison to the remaining 16 studies in which implants placement was after radiotherapy.

**Conclusions:**

Dental implants may be affected by radiotherapy especially when they are placed in maxilla, in grafted bone, or after radiation, however, they remain a functional option for rehabilitation of head and cancer patients. More Prospective cohort studies and randomized controlled trails are still needed to draw more evidence based conclusions.

** Key words:**Dental implants, implant survival, radiotherapy, head and neck cancer.

## Introduction

Head and neck cancer is common, it accounts for more than 550,000 cases annually worldwide (1). It is the sixth prevalent cancer site with a survival rate of 50% over 5 years (2). Although the survival rate in head and neck cancer remained unchanged during the past few decades (3), there is evidence that mortality rates have decreased over the last 20 years (4). Treatment modalities include a combination of surgery and radiotherapy. Surgery may cause anatomical alterations, and radiotherapy may result in oral mucositis, xerostomia, irradiation caries, fibrosis of blood vessels and soft tissues, and reduction of bone-healing capacity. Atrophied and erythematous mucosa and the condition of jaw bones render the placement of removable prosthesis a challenging procedure; thus, failure to restore satisfactory mastication usually adds to the overall morbidity of cancer therapy and results in decreased quality of life of head and neck cancer patients. The use of dental implants for rehabilitation offered many benefits over the conventional tissue-born prosthesis. These benefits include improved retention, mastication, and patient acceptance (5), however, dental implants rehabilitation is complex and it was considered a contraindication in the past for irradiated patients.

Radiation causes injury to the remodeling system by damaging osteoclasts and decreasing the proliferation of bone marrow, collagen, and blood vessels. Vascular injury shows as hyperemia followed by endarteritis and decreasing microcirculation. The bone marrow become hypocellular and hypovascular and shows signs of marked fibrosis and fatty degeneration. It is believed that the irradiated hypocellular, hypovascular and hypoxic tissue is the main cause of failures in dental implants osseointegration (6).

The aim of this article is to explore the literature between 2000 and 2014 of the effect of radiotherapy on dental implant survival in head and neck cancer patients with consideration of the role of implant location, bone augmentation, dose of radiation and timing of implant placement.

## Material and Methods

The protocol of the study was conducted according to the Preferred Reporting Items for Systematic Review and Meta-Analyses (PRISMA) statement (7). The review was conducted to answer the following specific question: “Is the survival of dental implants affected by radiotherapy?” 

A search in the electronic databases of the National Library of Medicine (http://www.ncbi.nlm.nih.gov) was conducted for the articles published between January 2000 and December 2014 about the effect of radiotherapy on survival of dental implants. The following keyword were used specifically: “radiotherapy”, “dental implants”, “survival”, and “head and neck cancer”. Boolean operator (AND) was used to combine searches. The abstracts found and their reference lists were reviewed to identify potentially pertinent articles. studies reporting outcomes of dental implants in irradiated and non-irradiated patients were considered eligible for inclusion. Exclusion criteria consist of studies on craniofacial implants or on animal subjects.

The review process was conducted by two independent reviewers through screening the publication titles and abstracts. Any disagreement between the reviewers was resolved by discussion and / or consultation of additional review author when necessary. Full manuscripts for included studies were obtained and evaluated further. The evaluation process considered the survival rates of dental implants in irradiated patients and other related factors which include; site of implant placement, bone augmentation , dose of radiation, and whether the placement of implant was before radiotherapy (primary) or after radiotherapy (secondary).

## Results

The database search yield 27 results and the manual search provided 6 additional publications. 15 articles were excluded after review of the title and abstract. The exclusion criteria included studies on craniofacial implants, and those comparing the survival of different implant surfaces. The 18 relevant results were original research articles based on clinical trials, case-control studies, case series and case reports related to the survival of dental implants in irradiated patients with head and neck cancer, and published in English. The investigated studies reported treatment of 1175 patients with 5245 implants, 2100 of them were controls in-serted in non-irradiated bone. Most of the studies were retrospective in nature. The quality and the level of evidence is generally low. A summary of all studies included in this review is shown in  Table 1. The result of the evaluation process was as follows:

**Table 1:**
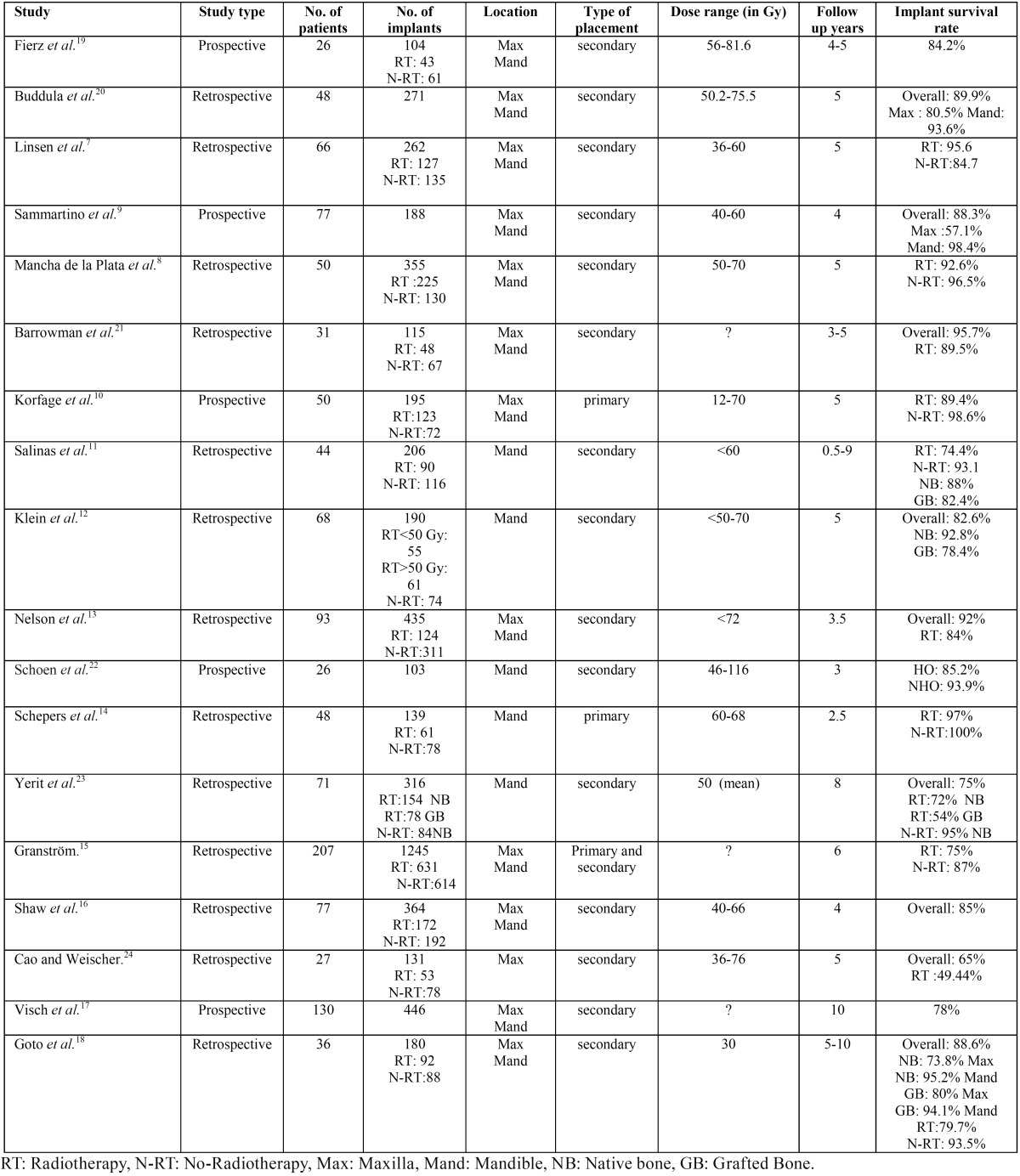
A summary of the current literature of dental implants survival in irradiated jaws.

-Survival of dental implants in irradiated bone

Twelve studies reported that radiotherapy have no significant impact on the survival of dental implants (8-19). The survival rate reported ranged between 74.4% (12) and 97% (15). However, 6 studies cautioned that dental implants survival may be affected negatively by radiotherapy (20-25). The survival rates reported were as low as 49.44% (25). Follow up periods ranged between 2.5 and 10 years.

-The role of site of implant placement in survival

The role of site of implant placement in survival have been discussed in 10 studies. 7 of them reported that implants can osseointegrate with greater success in the irradiated mandible than irradiated maxilla (10,12,13,17-19,21). A single study (25) in which dental implants were inserted exclusively into the irradiated maxilla reported a very low survival rate (49.44%). Contradictory findings assuming favorable survival of dental implants in the irradiated maxilla were reported (16). One study (8) concluded that implant survival is not significantly influenced by location (maxilla or mandible).

-The role of bone augmentation in implant survival

The survival rates of dental implants in irradiated native bone versus irradiated grafted bone were compared in 5 studies, all of them reported that irradiated grafted bone showed a significantly reduced dental implant survival rate in comparison to irradiated native bone (12,13,17,22,24). Survival rates reported in native bone ranged between 72-92.8%, while in grafted bone this range was between 54-82.4%. The success rate of implants placed in vascularized bone grafts reached up to 82.4% (12) in comparison to implants placed in non-vascularized bone grafts (54-78.4%) (13,24).

-The role of radiation dose in implant survival

The radiation doses reported in the investigated literature ranged between 30-116 Gy. This systematic review has shown that 6 out of 18 studies in which radiation doses exceeded 70 Gy (20-25) reported lower survival rates of dental implants in comparison to studies in which radiation doses were ≤70Gy (8-19).

-The role of timing of implant placement in survival

Only 2 studies (11,15) reported exclusively primary placement of dental implants (before the initiation of radiotherapy). The survival rates reported were 89.4% by Korfage *et al.* (11) and 97% by Schepers *et al.* (15). Noteworthy, the reported survival rates in primary placement are higher in comparison to that in secondary placement.

## Discussion

-Survival of dental implants in irradiated bone

There is a conflict in the evidence that support dental implant survival in head and neck cancer patients. In a recent review by Chambrone *et al.* (26), it was concluded that radiotherapy was associated with higher rates of implant failure in the majority of individual studies. Another meta-analysis of the current literature between 2007 and 2013 and the literature of the years 1990-2006 conducted by Schiegnitz *et al.* (5) revealed that in the current literature (2007-2013) there is no statistically significant difference in implant survival between non-irradiated native bone and irradiated native bone, however, the literature of the years 1990-2006 showed a significant difference in implant survival between non-irradiated and irradiated patients with a higher implant survival in the non-irradiated bone. These findings along with the findings of this study indicate that fundamental changes regarding implant survival in irradiated patients have taken place which can be explained by the recent advancements in implants therapy including the three-dimensional planning, guided implant surgery, technical improvements in implant surface features and shifts in treatment concepts. As a result, dental implants now seem to be a favorable treatment option for oral rehabilitation of patients with head and neck cancer with a history of radiation therapy (5), however, The patients should be informed in advance about complications associated with implants insertion when there is a history of irradiation (27).

-The role of site of implant

The implant location was reported to significantly affect its survival rate (21). Despite that mandible is considered the area which is most susceptible to osteoradionecrosis, there were cumulative reports in the literature that implants can osseointegrate with greater success in the irradiated mandible than irradiated maxilla. Maxillary implants may exhibit 496% greater risk of failure than mandibular implants (26). The results in this review indicate that studies on dental implants in irradiated maxilla are scarce. It was considered that a meta-analysis to examine the influence of the jaw region on implant survival was not feasible due to lacking data (5,27). The higher survival rate of dental implants in the mandible was attributed to the anatomy (28), and the higher bone density which provide better initial primary stability for the implant (29). Even in the same arch, the survival rate may differ from one location to another. Roumanas *et al.* showed that implants located in maxillary anterior sites demonstrated statistically significant differences in annual bone height changes compared with maxillary posterior sites (30). Moreover, dental implants can be placed in the anterior mandible with a high degree of predictability because most patients irradiated for head and neck tumors do not receive radiation to the symphyseal region (31).

-The role of bone augmentation

Ablative surgery may imply the use of bone grafts for reconstruction. These grafts may be vascularized free flaps or non-vascularized bone grafts. Rehabilitation necessitate the use of dental implants on native and grafted bone as well. The findings of this study coincide with earlier studies which considered that irradiated grafted bone showed a significantly reduced dental implant survival rate in comparison to irradiated native bone. Moreover, dental implant survival rate for free flaps was significantly greater than that in non-vascularized bone grafts (5,28). The combination of grafted bone with radiotherapy is considered a negative prognostic factor of implant survival. The lower survival rate of dental implants in the grafted bone may be reasoned by the differences in bone quality, bone volume, and revascularization of grafted bone when compared with the original local bone. Hence, implant placement in native bone should be preferred. The options available for maxillofacial reconstruction include non-vascularized bone grafts and vascularized bone flaps. In non-vascularized bone grafts the bone is revascularized by the process of creeping substitution. Cortical bone can be harvested from iliac crest, split calvarium, and rib. Non-vascularized bone grafts are restricted to defects lesser than 5cm long (32). Radiotherapy was considered a contraindication for using non-vascularized bone grafts because the soft tissue bed will be less desirable due to hypovascular, hypoxic, and hypocellular environment (33). Additionally, non-vascularized bone grafts shows less density and greater bone resorption. Vascularized bone flaps are considered the gold standard in oncologic reconstruction. In comparison to non-vascularized bone grafts, bone flaps can be used in large segmental bone reconstruction and can tolerate radiation therapy without resorption, fracture, or extrusion. Vascularized bone flaps can be either pedicled or free bone flaps. The use of pedicled bone flaps, such as the pectoralis major muscle with rib or sternal bone and the trapezius muscle with scapula is primarily of historic significance. Disadvantages of these tow flaps include limited ability to shape and configure both the soft tissue, and the bony flap components to fit the defect, restricted reach, and limited availability of bone. Therefore, free bone flaps (fibula free flap, iliac crest free flap, scapular free flap, and radial forearm free flap) are considered primary options for reconstruction. Fibula free flap is frequently used choice for mandibular reconstruction. The 22-25 cm of bone that can be harvested from fibula permit the reconstruction of near total mandibular defects. The double barrel approach may allow increasing the height of bone in anterior parts of the mandible, while Laterally, the width of a single fibular segment closely approximates the height of the native mandible (34). The iliac crest free flap can provide both of cortical and cancellous bone in generous amounts. The curved contour of iliac bone is ideal for lateral mandibular reconstruction. Dental implants can be reliably accommodated when the iliac crest bone is harvested as a full-thickness bicortical rather than as a partial-thickness unicortical bone flap (32). Scapular free flaps are used for reconstruction of the anterior mandible in patients who are not candidates for fibular free flaps (35), however, a major disadvantage of the scapular bone is that it is often quite thin so it does not always provide enough bone stock for placement of dental implants. In radial forearm free flap, limited thickness of bone can be harvested due to donor site morbidity, therefore, placement of dental implants in radial free flap is less reliable than other bone flaps. To sum up, the fibula and iliac crest free flaps render the best bone amounts for osseointegration, while the scapula and radial forearm do so less reliably.

-The role of radiation dose

There is no consensus in the literature about the threshold dose of radiation that may affect dental implant survival. Osteoradionecrosis and implant survival may depend on the dose of radiation. It was reported in the literature that the risk of osteoradionecrosis increase with doses that exceed 50Gy (18), 60Gy (36), 65Gy (37), and 70Gy (6). Soft tissue necrosis can take place with doses lesser than 50Gy, and injury to salivary glands can occur with doses of even lesser than 20Gy (6,38). The risk and severity of osteoradionecrosis is related to radiation dose, volume of irradiated tissue, and to the dental health of the patients (39). It was suggested that Prior to implant placement, consultation with the radiation oncologist is valuable to obtain radiation dose distribution that may assist planning the best locations for implants insertion (40). Several authors reported better survival rates with lower doses of radiation doses (18,27,28,41). Nevertheless, low incidence of small-dose radiation therapy studies preclude confirming such findings.

-The role of timing of implant placement

The timing of implant placement whether before or after radiotherapy is a very important issue which can affect the success or failure of osseointegration. This issue is widely debated and there is no scientific evidence for the optimal implant placement time until now. Immediate implant insertion before radiotherapy and during the ablative tumor surgery, is referred to as primary placement, while placement after radiotherapy regardless of the time interval is referred to as secondary placement. Primary placement was advocated in order to achieve osseointegration prior to the damaging effects of radiotherapy and to avoid additional surgery for oral rehabilitation (15,42). Correct placement is now facilitated by the recently introduced concepts of computer-guided implants which improved the identification of the ideal implant location during surgery (43). However, Primary placement may lead to interference with or delay of the oncological therapy including radiotherapy and is not always available to patients in the hospital settings (40). Secondary placement allows evaluation of the postsurgical status of the patients and the cancer prognosis. When secondary placement is considered, the patient by this time is aware of the altered physical and physiological state due to oncologic treatment, accepts the shortcomings and is psychologically prepared to extended treatment and rehabilitation (44). Nooh (28) reported 92.2% survival rate of dental implants before radiotherapy and 88.9% after radiotherapy. However, statistical verification is not possible because of the marked difference in the number of the studies in each group. There were insufficient data regarding the time interval of implant placement after radiation therapy. Typically dental implants are placed after a delay of 6 months after radiotherapy (27,44,45), but it is still unknown whether longer delays are beneficial. It have been reported that there is no significant difference between the survival rates of implants placed ≥12 months and ≤ 12 months after radiotherapy (18,24). In a recent systematic review of observational studies it was found that a higher risk of failure may result from placement of dental implants shorter than 12 months after radiotherapy, however, there is no evidence from clinical trials to verify this risk (46). Granstrom (47) reported that implant placement occurring decades after radiation therapy is more deleterious than early placement because there is a reduction in healing potential which can be explained by the progressive endarteritis which is known to increase with time.

## Conclusions

Survival rates of dental implants may be affected negatively by radiotherapy, however, they can osseointegrate and remain functionally stable and hence they can be considered a viable treatment option for rehabilitation and improvement of the quality of life of head and neck cancer patients. Maxillary sites, use of bone grafts and higher radiation doses are negative prognostic factors. Prospective cohort studies and randomized controlled trails are still needed to draw more evidence based conclusions about survival of dental implants in head and neck cancer patients.
